# Supplementary material for: Silicon Wafer CMP Slurry Using a Hydrolysis Reaction Accelerator with an Amine Functional Group Remarkably Enhances Polishing Rate
Source: Nanomaterials (Basel). 2022 Nov 4;12(21):3893. doi: 10.3390/nano12213893 (PMC9656662; doi:10.3390/nano12213893)
Supplement: Supplementary file 1 [file nanomaterials-12-03893-s001.zip › nanomaterials-1993560-supplementary.pdf]

Supplementary Materials

Silicon Wafer CMP Slurry Using a Hydrolysis Reaction Accelerator with an Amine Functional Group Remarkably Enhances Polishing Rate

Jae-Young Bae[1], Man-Hyup Han[2], Seung-Jae Lee[3], Eun-Seong Kim[2], Kyungsik Lee[3], Gon-sub Lee[3], Jin-Hyung Park[4], and Jea-Gun Park[2,3*]

[1] Department of Energy Engineering, Hanyang University, Seoul 04763, Korea
[2] Department of Nanoscale Semiconductor Engineering, Hanyang University, Seoul 04763, Korea
[3] Department of Electronic Engineering, Hanyang University, Seoul 04763, Korea
[4] UB Materials Inc., Yongin 17162, Korea
* Correspondence: parkjgl@hanyang.ac.kr

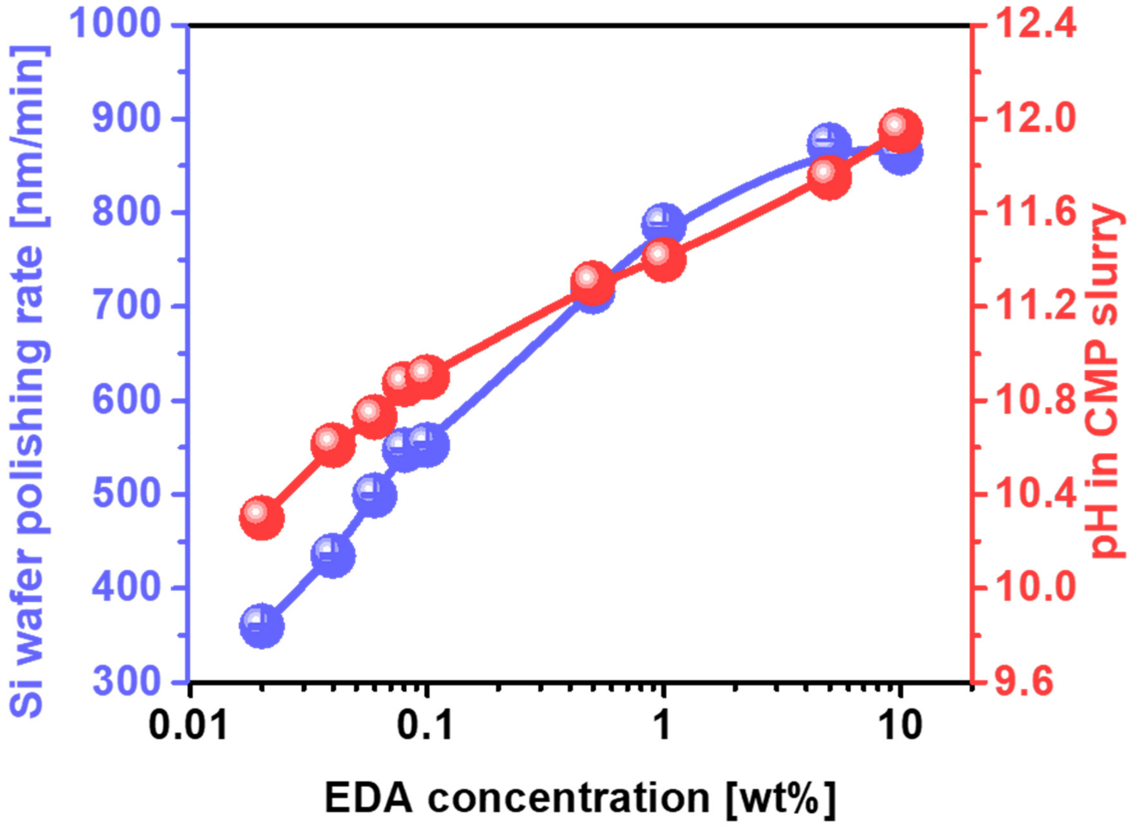

Figure S1. The dependency of Si wafer polishing rate and pH on EDA concentration.

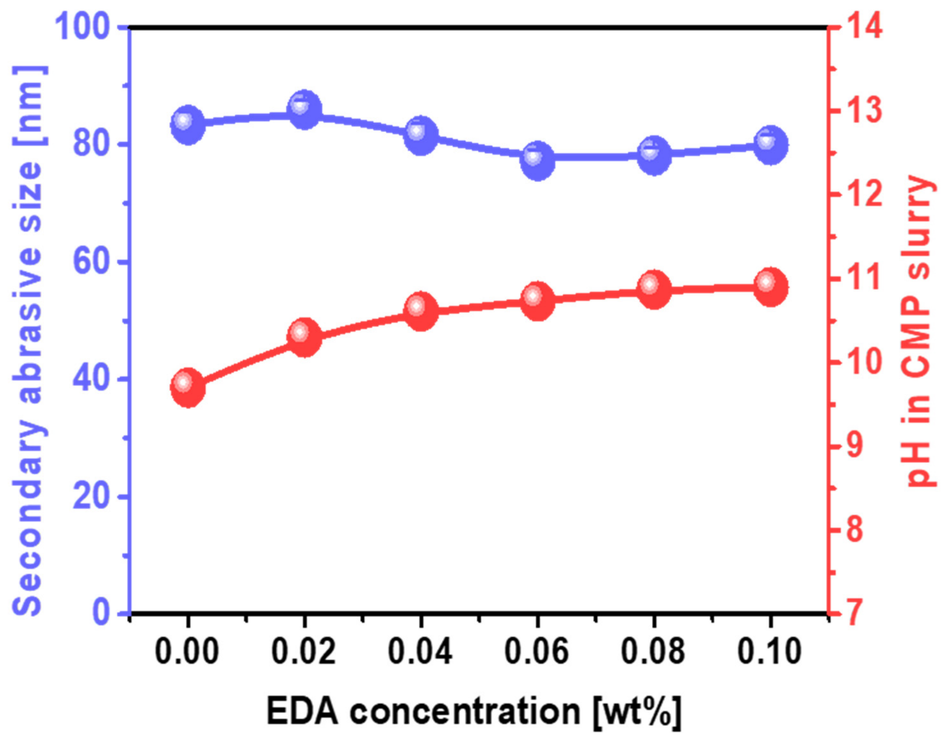

Figure S2. The secondary colloidal silica abrasives size and pH depending on EDA concentration in the Si wafer CMP slurry. The secondary colloidal silica abrasives size was independent of the EDA concentration. The pH of slurry slightly and root-squarely the EDA concentration.

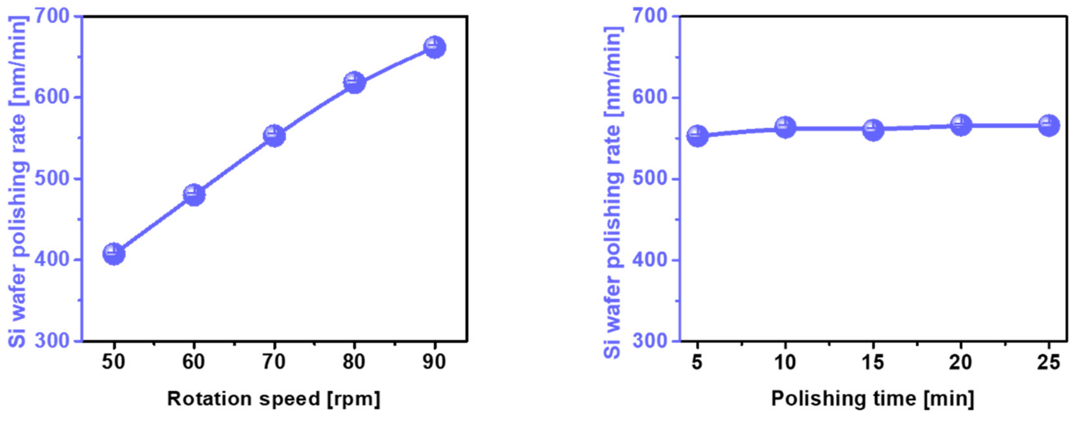

(**a**) (**b**)

Figure S3. Dependencies of the Si wafer polishing rate on the head and table rotation speed and polishing time. (**a**) Effect of the rotation speed on the Si wafer polishing rate and (**b**) effect of the polishing time on the Si wafer polishing rate.

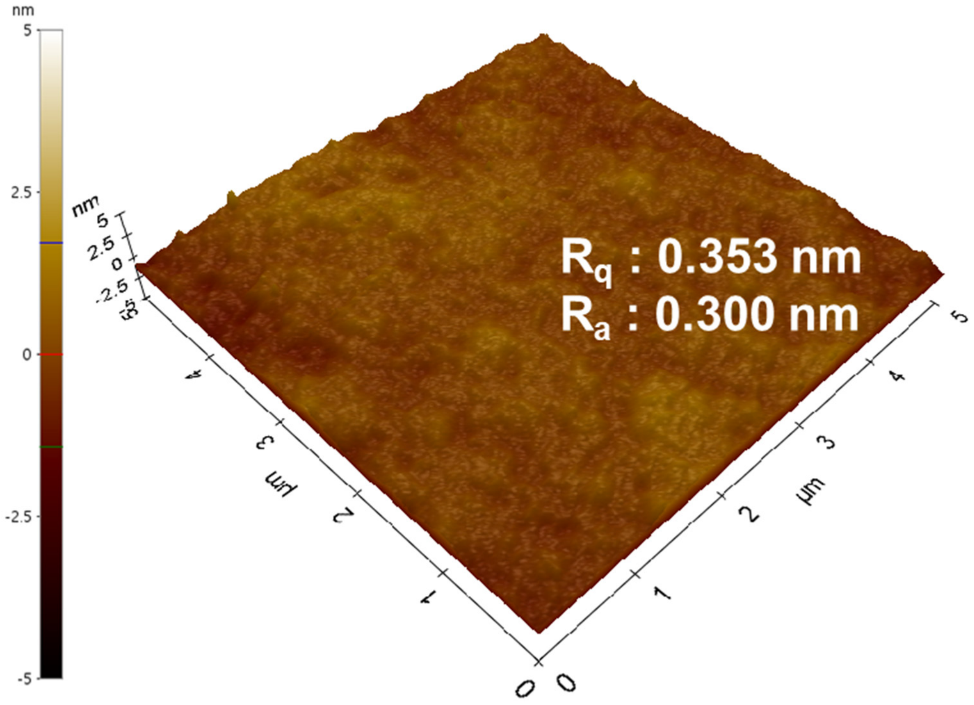

Figure S4. Surface roughness of the polished Si wafer after CMP using the slurry including 60-nm-diameter colloidal silica abrasives of 1 wt% and EDA of 0.10 wt%. The R_q (i.e., Root Mean Square Roughness) of the polished Si wafer was 0.353 nm by using AFM (5μm × 5μm in scanning area).

Table S1. Si wafer polishing rate comparison for the Si wafer CMP slurry using NaOH, KOH, and commercial Si wafer CMP slurries.

List	Si wafer CMP Slurry Properties					
	Abrasives	Accelerator	pH	Conductivity [uS/cm]	Polishing-rate [nm/min]	Manufacturer
Our work	Silica @ 60 nm 1 wt%	Ethylenediamine 0.10 wt%	10.90	379.8	552.8 ± 2.15	-
Slurry using NaOH	Silica @ 60 nm 1 wt%	NaOH 0.125 wt%	10.90	501.2	177.1 ± 1.07	-
Slurry using KOH	Silica @ 60 nm 1 wt%	KOH 0.069 wt%	10.90	490.8	193.2 ± 1.07	-
Commercial slurry A	Silica @ 70 nm 1 wt%	Non-disclosed	10.29	1065.0	372.3 ± 2.68	Fujimi Inc.
Commercial slurry B	Silica @ 15 nm 1 wt%	Non-disclosed	10.52	826.0	316.7 ± 1.61	HANA Materials Inc.

Table S2. Si wafer polishing rate comparison for the Si wafer or polysilicon CMP slurries via hydrolysis reaction with amine functional group. Note that a higher rotation speed, head pressure, and a harder pad leads to a higher Si wafer polishing rate. Our work presented a remarkably high Si wafer polishing rate at a relatively low rotation speed (i.e., 70 rpm), a low head pressure (i.e., 4 psi), and a softer pad (i.e., Suba 600).

List	Slurry Compositions				CMP Conditions					Reference
	Abrasives	Hydrolysis reaction accelerator	pH	Dilution ratio [slurry:DIW]	Polishing rate [nm/min]	Head pressure [psi]	Rotation speed [rpm]	Flow rate [mL/min]	CMP pad	
Si wafer CMP	colloidal silica (35 wt%)	organic bases[1] as pH titrant (1.5 wt%)	10.5-12	1:15	~ 700	58	100	50	non-disclosed	[29]
Si wafer CMP	colloidal silica @ 30 nm (non-disclosed)	poly amine (Non-disclosed)	9.5-11.3	1:15	1450	17.4	60	300	CP-P-32 (hard pad)	[30]
polysilicon CMP	colloidal silica @ 30 nm (1.3 wt%)	tetramethylammonium hydroxide (0-0.8 wt%)	6.8-11.8	-	261.3	4	30	210	IC1000 (hard pad)	[18]
polysilicon CMP	colloidal silica @ 50 nm (10 wt%)	arginine (0-2 wt%)	3-11	-	~ 450	4	75	200	IC1000 (hard pad)	[27]
polysilicon CMP	abrasive-free	guanidine carbonate (1 wt%)	4-11	-	250	4	75	200	IC1000 (hard pad)	[28]
Si wafer CMP	colloidal silica @ 60 nm (1 wt%)	ethylenediamine (0-0.1 wt%)	9.7-10.9	-	552.8	5.7	70	100	Suba 600 (soft pad)	Our work

[1] Organic bases; ethanolamine, diethanolamine, ethylenediamine, diethylenetriamine, triethylenetetramine, and aminoethylethanolamine

Table S3. Production price for hydrolysis reaction accelerator with amine functional group.

List	Information		
	Chemical structure	Chemical grade	Production price
Ethylenediamine	$C_2H_4(NH_2)_2$	for synthesis	$75.60 / L
Diethylenetriamine	$HN(CH_2CH_2NH_2)_2$	for synthesis	$85.70 / L
Triethylenetetramine	$(CH_2NHCH_2CH_2NH_2)_2$	for synthesis	$117.00 / L
